# Prospective comparison of switches in biomarker status between primary and recurrent breast cancer: the Breast Recurrence In Tissues Study (BRITS)

**DOI:** 10.1186/bcr2771

**Published:** 2010-11-08

**Authors:** Alastair M Thompson, Lee B Jordan, Philip Quinlan, Elizabeth Anderson, Anthony Skene, John A Dewar, Colin A Purdie

**Affiliations:** 1 Department of Surgery and Molecular Oncology, Ninewells Hospital and Medical School, Dundee, DD1 9SY, UK; 2 Department of Surgical Oncology, MD Anderson Cancer Center, 1515 Holcombe Boulevard, Houston, TX 77030, USA; 3 Department of Pathology, Ninewells Hospital and Medical School, Dundee, DD1 9SY, UK; 4 AstraZeneca, 15 Stanhope Gate, London, W1K 1LN, UK; 5 Department of Surgery, Royal Bournemouth Hospital, Castle Lane East, Bournemouth, Dorset, BH24 4AX, UK; 6 Department of Oncology, Ninewells Hospital and Medical School, Dundee, DD1 9SY, UK

## Abstract

**Introduction:**

Immunohistochemistry of primary breast cancer is routinely used to guide changes in therapy at the time of relapse. Retrospective reviews suggest that the estrogen receptor (ER), progesterone receptor (PR) and human epidermal growth factor receptor type 2 (HER2) receptor may differ between the primary and loco-regional recurrence or distant metastases. The Breast Recurrence In Tissues Study (BRITS) was a large, multicentre, prospective study to examine changes in ER, PR and HER2.

**Methods:**

Matched primary and recurrent breast cancer tissue samples were prospectively collected from 205 women attending 20 institutions. Central laboratory immunohistochemical analysis of core biopsies and tissue microarrays of ER and PR using the Allred and Quickscore methods and HER2 (confirmed by fluorescence in situ hybridisation (FISH) for HER2 2+) were performed.

**Results:**

From 205 consenting women, 18 (8.8%) did not have recurrent disease on biopsy, 35 were ineligible, 13 had insufficient paired tissue and 2 were excluded for safety reasons. Paired samples from 137 women, mean age 62.6 years (range 27-87 years), 83/137 (60.6%) postmenopausal with a median 92.2 months (range 5-327 months) from primary to recurrence and 88 (64.2%) as locoregional recurrence were successfully analysed. A switch in receptor status, in either direction, by Allred score, was identified for ER in 14 patients (10.2%; P = 0.983 Wilcoxon sign rank test), PR in 34 (24.8%; P = 0.003 Wilcoxon sign rank test) and HER2 in 4 (2.9%; P = 0.074 Wilcoxon sign rank test). There was no difference between locoregional or distant recurrence in the proportion who switched. The switch in receptor status led to a change in the subsequent treatment plan for 24 patients (17.5%).

**Conclusions:**

This prospective study confirms retrospective evidence that the management of relapsed breast cancer should include confirmatory tissue sampling and identify switches of ER, PR or HER2 which change therapeutic management for one in six patients.

## Introduction

The management of recurrent breast cancer requires evidence-based approaches [1] since the median survival in patients with overt metastatic disease is 20 months [2]. Current opinion supports reassessment of estrogen receptor (ER), progesterone receptor (PR), and human epidermal growth factor type 2 (HER2) receptor in tumor tissue at the time of diagnosis of relapse to tailor appropriate therapy for each patient [3,4]. This is based largely on retrospective evidence that loss of ER in recurrent breast cancer [5] is an established predictor for poor response to endocrine therapy [6].

Historically, ER, PR, and HER2, where available from the primary cancer, have been used to direct subsequent therapy, assuming no change in the biological features of the recurrent disease compared with the original primary; this approach is no longer considered tenable [3,4]. Although molecular approaches have been used with mixed results to compare primary and recurrent breast cancer [7-9], such transcriptome approaches have yet to be validated in the context of recurrent disease.

Studies of paired samples of the primary tumor and locally/regionally recurrent or distant metastases suggest that tumor receptor status may be discordant in a significant proportion of patients: 18% to 54% for ER, 36% to 54% for PR, and 3% to 22% for HER2 in both retrospective series [6,10-14] and small prospective series [15-17].

Routine diagnostic histopathology and immunohistochemistry (IHC) of recurrent breast cancer for ER, PR, and HER2 (with fluorescence *in situ *hybridization [FISH] testing of HER2 where appropriate) may be a pragmatic solution to ensure that the patient actually has recurrent breast cancer [17] and to guide further patient therapies. The Breast Recurrence In Tissues Study (BRITS) sought to establish the value of these established diagnostic approaches in a prospective, multicenter evaluation. The BRITS set out to quantify the percentage of tumors that changed receptor status (positive to negative or negative to positive) for ER, PR, and HER2 expression between the original and recurrent tumor in women with breast cancer and to determine the proportion of patients in which a switch in ER, PR, or HER2 led to a change in the subsequent treatment plan.

## Materials and methods

**Figure 1:**
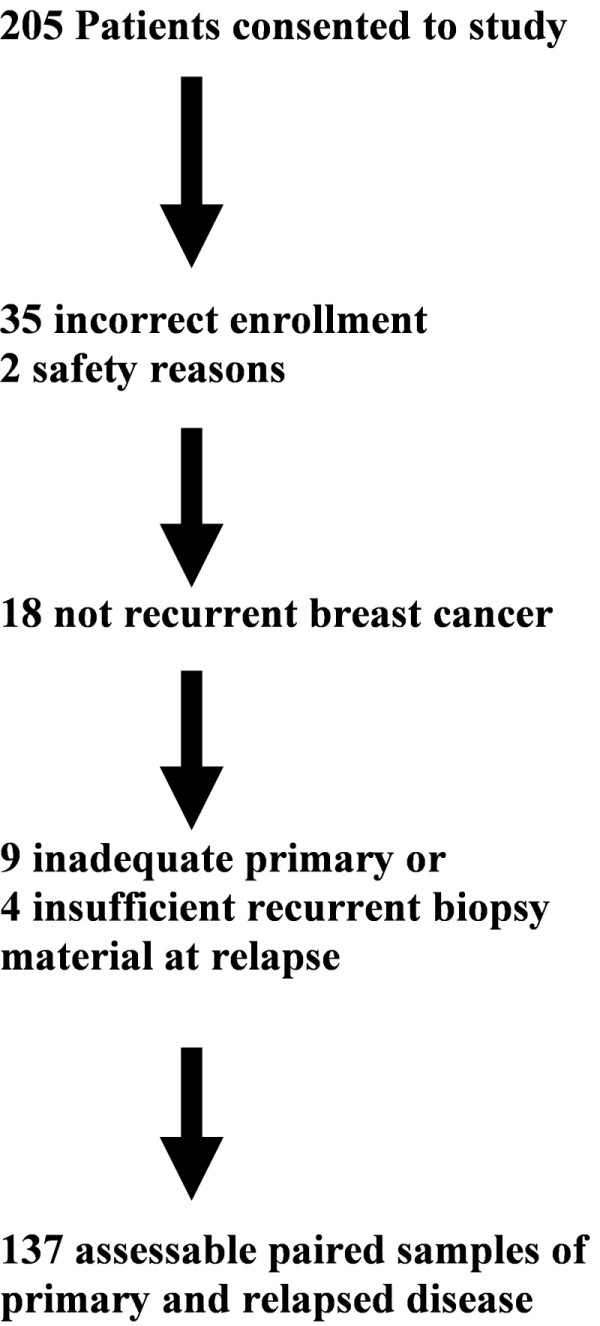
**Consort diagram of the Breast Recurrence In Tissues Study (BRITS)**.

Women who had a history of invasive breast cancer and who were willing to consent to biopsy of recurrent disease (locoregional or distant metastasis) were invited to participate in the BRITS during 2007-2008 at 20 secondary care sites in the UK. Multicenter ethics permission for the BRITS was obtained through the North Glasgow Ethics Research Committee. Breast cancer in a conserved breast was pragmatically considered to be recurrence rather than a new primary cancer, but contralateral primary invasive cancer was excluded. Two hundred five women provided written informed consent to prospectively participate in order to supply 137 good-quality paired tumor samples (Figure 1).

Patients were required to have available a formalin-fixed paraffin-embedded (FFPE) tumor from both the primary cancer and the recurrence to be fully evaluable. FFPE tissue at the time of recurrent breast cancer was biopsied (as a core biopsy or resected tissue) and diagnostic review was conducted by the local pathologist to confirm the presence of invasive breast cancer. FFPE from the primary cancer was subsequently retrieved from the local pathology department, paired with the prospectively collected recurrent breast cancer FFPE block, and sent for central specialist pathologist review by CAP and LBJ.

Central pathology review comprised new full-face hematoxylin and eosin (H&E) section of all specimens to confirm the presence of sufficient, suitably fixed invasive breast cancer in both primary and recurrent specimens. All patients had a full-size tissue block from the original primary cancer; excisional tissue was available from the recurrent disease of 100 patients (73%), and core biopsy only was available from 37 women (27.0%). Blocks were marked for tissue microarray (TMA) construction of 6 × 0.6 mm cores of invasive cancer TMA (Beecher Instruments, Sun Prairie, WI, USA). Thus, in 100 patients, the primary and recurrent disease was constructed into TMA blocks, whereas in 37 patients, the TMA of the primary was compared with full-face sections of core biopsies of the recurrent disease.

### Immunohistochemistry

Immunohistochemical staining was carried out on 4-μm sections of FFPE TMAs or full sections of cores with the mouse monoclonal anti-ER-alpha antibody 6F11 1:200 (Novocastra Laboratories Ltd, Newcastle-upon-Tyne, UK), PR antibody clone 16, 1:800 (Novocastra Laboratories Ltd), and a mouse monoclonal anti-HER2 antibody CB11 (Novocastra Laboratories Ltd) as primary antibody. Negative controls (lacking primary antibody) were performed for all samples stained. Antigen retrieval for ER and PR was carried out using a microwave/pressure vessel followed by processing on a DAKO TechMate™ 500 Plus autostainer (Dako, Glostrup, Denmark) with the DakoREAL™ detection system (an indirect streptavadin/biotin method using anti-mouse antibody) and visualized with horseradish peroxidase and diaminobenzadine.

ER and PR status was assessed by means of two methods: primarily the Allred score [18], which is used as a current worldwide standard for ER and PR reporting, and the 'Quickscore' method, which examines both the intensity and proportion of cells stained [19]. With the Allred score, tumors scoring 0 or 2 were regarded as negative from the point of view of endocrine therapy, whereas cases scoring at least 3 were regarded as positive. HER2 scoring was carried out with the standard negative (0), negative (1+), equivocal (2+), and positive (3+) system [20].

TMA and full-section immunohistochemical staining conducted in this laboratory (as described) demonstrates concordance of IHC scoring for ER, PR, and HER2 [21]. Indeed, just two 0.6-mm cores, rather than the six used here, may be sufficient to represent staining seen on an entire histological section, even for markers generally thought to be heterogenous [21]. For all IHC, investigators were blinded, where possible, to the clinical data. Clearly, core biopsy samples could be from recurrent breast cancer only; cases that were difficult to score were subject to consensus reporting by LBJ and CAP on a multi-headed microscope.

### HER2

HER2 amplification was assessed in the nationally accredited regional cytogenetics laboratories as previously described [20]. Briefly, FISH was conducted using a locus-specific probe (Qbiogene Inc., MP Biomedicals Europe, Illkirch, France) on full-face or core biopsy sections that were 4 μm thick. All HER2 slides were viewed using an Olympus BX51 epi-fluorescence microscope (Olympus, Tokyo, Japan) equipped with DAPI, SpectrumGreen™, and SpectrumOrange™ filter cubes. Images were captured by means of Applied Spectral Imaging acquisition software (Edingen, Germany). The ratio of orange (HER2) to green (CEP17) signals (PathVysion Her2 assay; Vysis, Inc., Downers Grove, IL, USA) was calculated for all of the cases studied. A ratio of at least 2.0 was counted as amplified. At least 10 non-overlapping nuclei were scored for amplified tumors.

HER2-positive cancers were thus defined as those that were IHC 3+ (all of which were also amplified by FISH) or HER2 2+ and FISH-amplified (all but one of the HER2 2+ cancers had amplification using FISH). Redefinition of the cutoff for amplification of at least 2.2 did not alter the categorization of the cancers as no amplified tumors had a FISH amplification ratio of between 2.0 and 2.2.

### Statistical analysis

Sample size was determined prior to commencement of the study by using the percentage of subjects switching ER status (ER-positive to ER-negative or ER-negative to ER-positive) from primary to recurrence as the primary endpoint. On the basis of published retrospective data available at the time of study design (including [6,10,12]), a conservative assumption was made that if 10% of subjects switched ER status between the initial biopsy and the biopsy taken at recurrence, a sample size of 139 fully evaluable subjects would yield a 95% confidence interval (CI) with a width of ± 5% to detect a significant difference in ER.

The statistical significance of the change in ER, PR, and HER2 score was analyzed by means of one sample student test (95% CIs and *P *value). To allow for the potential that data were not normally distributed, the *t *test was replaced with the Wilcoxon sign rank test, median differences, the associated CIs, and the *P *value. Associations between the original sample score and the change in the ER, PR, and HER2 expression scores, as measured by IHC or FISH between the original and recurrent samples, were considered using the Pearson correlation coefficient (if data were assumed to be continuous) and, in keeping with clinical decision making (considering ER, PR, and HER2 to be positive or negative), the Spearman correlation coefficient. The association between the switch in status of ER, PR, and HER2 and primary tumor size (defined in ordered categories), grade of tumor (1, 2, 3), node status, and the Nottingham Prognostic Index were investigated by the chi-square test (with degrees of freedom and the associated *P *value). The categories of each variable were summarized and presented as frequency and percentage.

Logistic regression analyses were performed to determine the association between switching status in ER, PR, and HER2, the time between diagnosis and recurrence and the number and type of previous treatments received for breast cancer. The differences between groups were summarized by the odds ratio (odds that the subject switched status with respect to an additional previous breast cancer treatment or an additional unit time between diagnosis and recurrence, respectively), associated 95% CIs, and *P *value.

## Results

From 205 women who consented to the BRITS (Figure 1), 18 (8.8%) did not have recurrent malignancy on biopsy (or indeed recurrence elsewhere), despite an initial clinical diagnosis of disease relapse. Nine patients had insufficient primary or recurrent tissue for study on review of the tissue blocks, 35 women were incorrectly enrolled, and 2 withdrew because of safety concerns (regarding biopsy). For the 137 women with paired primary and recurrent tissue samples, the mean age at disease recurrence was 62.6 years (standard deviation of 12.3 years), median age was 63.0 years, and range was 27 to 87 years. All but two women were Caucasian, and 83 out of 137 subjects (60.6%) were postmenopausal. The mean time to first recurrence of breast cancer following completion of primary therapy was nearly 8 years (93.2 months), and the mean time from original tissue sampling to the biopsy used for the BRITS was 9 years (106.7 months; range of 5 to 327 months). Previous therapies included endocrine therapy for 100 out of 136 (73%) (1 patient not known), previous chemotherapy for 62 (45.3%) (2 patients not known), and previous radiotherapy in 108 (78.8%) (1 patient not known). There were no significant differences in demographic features between the 137 women included in the analyses and the 68 women excluded.

Seventy-two out of 137 subjects (52.6%) had a lumpectomy and 47 (34.3%) a mastectomy at the time of the original primary diagnosis (7 patients not recorded). A further 11 subjects (8.0%) had a lumpectomy followed by completion mastectomy. The majority of patients had within-breast (44.5%) or regional soft tissue/locoregional (19.7%) disease recurrence (Table 1).

**Table 1:** Sites of breast cancer recurrence

Site of recurrent disease	Number of patients (total = 137)	Percentage of patients
Locoregional disease	88	64.2%
Distant soft tissues	16	11.7%
Other distant metastasis	33	24.1%

### Pathology type

For the original primary cancer pathology subtype, 96 out of 137 subjects (70.1%) had invasive ductal cancer, 23 lobular, 10 tubular/cribriform, 4 mucinous, and 2 medullary, and 2 had missing data. In addition, 84 subjects (61.3%) had *in situ *carcinoma: 74 (88.1%) ductal carcinoma *in situ *(DCIS) and 8 (9.5%) lobular carcinoma *in situ*. No recurrent cancers demonstrated a change in subtype.

### Changes in estrogen receptor and progesterone receptor

Central laboratory analysis of the original primary cancer was ER-positive by Allred score in 109 out of 137 subjects (79.6%), PR-positive by Allred score in 85 subjects (62.0%), and HER2-positive in 14 subjects (10.2%) (Table 2). Central laboratory analysis of the recurrent breast cancer demonstrated that the ER staining was positive in 101 (73.7%), PR was positive in 75 (54.7%), and HER2 was positive in 16 subjects (11.7%).

**Table 2:** Changes in estrogen receptor, progesterone receptor, and HER2 receptors

Receptor status of primary	Number (Percentage)	Allred status of recurrence	Number (Percentage)	Quickscore status of recurrence	Number (Percentage)
ER^+^	109 (79.6%)	ER^+^	98 (71.5%)	ER^+^	100 (73.0%)
		ER^-^	11 (8.0%)	ER^-^	9 (6.6%)
ER^-^	28 (20.4%)	ER^-^	25 (18.2%)	ER^-^	24 (17.5%)
		ER^+^	3 (2.2%)	ER^+^	4 (2.9%)
PR^+^	85 (62.0%)	PR^+^	63 (46.0%)	PR^+^	53 (38.7%)
		PR^-^	22 (16.0%)	PR^-^	32 (23.4%)
PR^-^	52 (38%)	PR^-^	40 (29.2%)	PR^-^	40 (29.2%)
		PR^+^	12 (8.8%)	PR^+^	12 (8.8%)
				
		HER2 status of recurrence	Number (Percentage)		
				
HER2^+^	14 (10.2%)	HER2^+^	13 (9.5%)		
		HER2^-^	1 (0.7%)		
HER2^-^	123 (89.8%)	HER2^-^	120 (87.6%)		
		HER2^+^	3 (2.2%)		

ER, estrogen receptor; HER2, human epidermal growth factor type 2; PR, progesterone receptor.

With the Allred scoring, a switch in receptor status (Table 2) was identified for ER in 14 patients (10.2%): from ER-positive to ER-negative in 11 patients (8.0%) and from ER-negative to ER-positive in 3 (2.2%). A change in PR was noted in 34 cancers (24.8%): PR-positive to PR-negative in 22 patients (16.1%) and PR-negative to PR-positive in 12 (8.7%) in the recurrent biopsy. An additional 3 patients (2.2%) became HER2-positive, and 1 patient went from HER2-positive to HER2-negative.

The histological sections stained for ER or PR and rescored using the Quickscore method blinded to the Allred score demonstrated a difference in endocrine receptor switch compared with the Allred score in 13 patients (Table 2): from ER-positive to ER-negative (2) or ER-negative to ER-positive (1) and a switch from PR-positive to PR-negative (10) but not PR-negative to PR-positive (0). In keeping with the literature [21-23], where full-face sections were available and compared with TMA data for ER and PR, no differences in scoring were identified.

### HER2

Few patients (14, 10.2%) were HER2-positive at the time of primary diagnosis; 4 patients (2.9%) gained (3 patients) or lost (1 patient) HER2-positive tumor staining (Table 2), although this failed to achieve statistical significance for a change in HER2 receptor scores (*P *= 0.074). Most patients ( > 80%) were HER2-negative on both occasions.

### Clinical impact

A switch in ER, PR, or HER2 receptor status (positive to negative or negative to positive) overall occurred in 34 out of 137 patients (24.8%). The combination of ER and PR was used to guide the use of endocrine agents. Thus, in the opinion of the accruing clinicians at the recruiting centers, this change in receptor status (in either direction) led to a change in the planned next treatment for the relapsed breast cancer in 24 out of 137 women (17.5%): 20 based on endocrine receptor and 4 based on HER2 receptor changes (Table 3).

**Table 3:** Therapy change based on receptor status in 24 patients

Primary	Recurrence	Primary PR	Recurrence PR	Primary ER	Recurrence ER	Primary HER2	Recurrence HER2	Therapy change based on
Excision	Core	7	8	7	8	Positive	Negative	HER2
Excision	Core	7	7	8	8	Negative	Positive	HER2
Excision	Core	7	6	8	8	Negative	Positive	HER2
Excision	Excision	0	0	7	0	Negative	Positive	ER and HER2
Excision	Excision	0	0	7	0	Negative	Negative	ER
Excision	Excision	0	0	3	2	Negative	Negative	ER
Excision	Core	7	3	6	0	Negative	Negative	ER
Excision	Excision	8	0	7	0	Negative	Negative	ER
Excision	Excision	0	0	8	0	Negative	Negative	ER
Excision	Core	2	2	7	0	Negative	Negative	ER
Excision	Excision	5	0	6	2	Negative	Negative	ER
Excision	Excision	0	0	3	0	Positive	Positive	ER
Excision	Excision	4	0	7	0	Positive	Positive	ER
Excision	Excision	0	0	0	7	Negative	Negative	ER
Excision	Excision	0	6	0	8	Negative	Negative	ER
Excision	Excision	0	0	0	7	Negative	Negative	ER
Excision	Excision	6	0	8	2	Positive	Positive	ER and PR
Excision	Excision	4	0	0	0	Negative	Negative	PR
Core	Excision	5	0	0	0	Negative	Negative	PR
Core	Core	6	0	8	7	Negative	Negative	PR
Excision	Excision	6	0	7	7	Negative	Negative	PR
Excision	Excision	7	0	7	7	Negative	Negative	PR
Excision	Excision	7	0	7	7	Negative	Negative	PR
Excision	Excision	4	0	6	5	Negative	Negative	PR

Comparison of primary and recurrent breast cancer progesterone receptor (PR), estrogen receptor (ER), and human epidermal growth factor type 2 (HER2) with documented reason for change in subsequent therapy is shown. Receptor status is summarized as negative (-) or positive (+).

Comparison between locoregional recurrence and distant metastasis failed to identify a significant difference for changes in ER, PR, or HER2 (Table 4). There was no significant difference between subjects who switched ER, PR, and/or HER2 status and the number and type of previous treatments (including endocrine treatments) for breast cancer, the time between diagnosis and recurrence, the tumor size, grade of tumor, node status, or the Nottingham Prognostic Index derived from the primary cancer.

**Table 4:** Change in receptor status by locoregional or distant recurrence

Receptor	**Locoregional recurrence**, number (percentage)	**Distant recurrence**, number (percentage)
Estrogen receptor	9/88 (10.2%)	5/49 (10.2%)
Progesterone receptor	22/88 (25.0%)	12/49 (24.5%)
HER2	2/88 (2.3%)	2/49 (4.1%)

HER2, human epidermal growth factor type 2.

## Discussion

In contrast to the diagnosis of primary disease, biopsy of relapsed, locoregional, or distant metastatic breast cancer is not widely established in routine clinical practice, despite evidence suggesting that such a biopsy may influence patient management [3-5,14,17]. The historical default position for therapeutic decision making has been based on the IHC of the primary tumor, and the IHC may or may not reflect the relapsed disease.

The BRITS reports the largest, prospective, multicenter study of primary versus recurrent locoregional or metastatic breast cancer to date. The demographic considerations of the patients participating were representative of women with symptomatic breast cancer at presentation [1]. However, despite commitment from 20 centers, 68 patients were lost from the 205 initially recruited, including 18 patients (8.8%) with clinical misdiagnosis of recurrent breast cancer, confirming that biopsy may be necessary to confirm disease recurrence in up to 10% of patients [17]. Despite the large size of this prospective study, two potential sources of patient bias remain. The relatively long duration between the primary and recurrent disease may favor the later recurrence of ER-positive HER2-negative breast cancer, reflected in the relatively large proportion of high ER-positive and low HER2-positive cancers. Second, tissue acquisition of breast, chest wall, or axillary disease (two thirds of patients in the current study) as part of therapeutic completion mastectomy and associated surgery may fit with routine clinical practice and be more readily achieved than biopsy of distant metastatic disease. However, no difference in receptor switch with respect to locoregional or distant site of recurrence was found (Table 3), confirming a large retrospective review [14]. In keeping with this, autopsy data comparing primary to multiple metastatic sites suggested that the metastases were consistent in ER, PR, and HER2 with each other, even if different (loss of ER or PR) from the synchronous primary cancer [8].

Through the use of a single central laboratory and consensual reporting by two specialist breast pathologists, the study methodology has sought to address a number of technical issues, including the choice of antibody, antibody staining conditions, and scoring systems that are recognized difficulties with IHC [22,23]. The differential fixation of resected primary cancer compared with core biopsy has demonstrated the greater ER and PR positivity in core biopsies [24]. Given that, in the BRITS, cores were predominantly from distant metastatic lesions, the better fixation of cores could overestimate the gain of ER (identified in less than 3%) or PR (identified in less than 9%) identified in a minority of patients (Table 2).

Intratumoral heterogeneity, potentially generating sampling errors for both the primary and the recurrence, cannot be excluded from consideration. However, multiple cores (up to six per primary and six per recurrence) in excess of the two generally held to be sufficient [21] were selected by a specialist breast pathologist (CAP or LBJ) to generate the TMAs, and ER and PR were shown to be consistent between TMAs using multiple 0.6-mm cores from each cancer and full sections [21,25]. Then, attempts were made to minimize technical variables as a source of bias in reporting this study. The use of FISH, which has also been validated on TMAs [26], to clarify the HER2 status of both the primary and recurrent disease by an accredited cytogenetics service should give confidence that the progression to HER2 amplification demonstrated here in 2.2% of cancers accurately reflects cytogenetic gain in the relapsed disease compared with the primary cancer.

We considered whether the use of the Allred score (0 to 8) [18], though in keeping with many current histopathology reporting conventions, might be less discriminatory (presenting an 'all or nothing' result) to detect changes in ER/PR expression than other scoring systems. The rescoring of the sections (with investigators blinded to the Allred score) by means of the Quickscore method (0 to 18) [19], which multiplies cell intensity by proportion, is generally consistent with the Allred score but did identify a small number of patients in whom the ER changes (3) or PR changes (10) were sufficiently different in the Quickscore methodology and consequently crossed the clinical cutoff for endocrine therapy. Thus, both quality assurance of histopathology and the scoring regimens used for immunohistochemical analyses may impact on the clinical direction provided by histopathological review of relapsed breast cancer.

### Changes in endocrine receptor status

Changes in ER and PR status, though of clinical significance in directing changes in therapy, were of borderline statistical significance regardless of which scoring system was used, despite the pre-study statistical power calculations based on the primary endpoint of change in ER. The changes were in keeping with a recent 25-patient prospective study [17] and the discordance between the primary and recurrent disease in up to 36% of patients for ER and up to 54.2% for PR, and gain was less common than loss of ER or PR (reviewed in [14]). Furthermore, any distinction between considering ER or PR switches in the context of locoregional or metastatic disease may now be less relevant in the light of autopsy studies [8]. However, patients with concordant ER and PR in the primary and relapsed tumor may have a significantly better post-recurrence survival than discordant paired samples [27]. Similar prospective clinical follow-up in the context of the BRITS was not planned.

### HER2

The gain in HER2 in three patients identified here confirms prospective evidence that acquisition of HER2 amplification suitable for therapeutic targeting is clinically important [17]. Despite interpretational difficulties and issues of tumor heterogeneity, most, but not all [13,28], retrospective series report discordance between the primary and relapsed disease, usually gain of HER2 [14,29] rather than loss of HER2 [14,30].

### Clinical implications

The inclusion of tissue acquisition and analysis into routine practice in the management of locoregional or distant metastasis carries implications for the treatment of relapsed breast cancer. While the time needed to obtain such a biopsy may raise patient concerns [17], avoiding misdiagnosis based on clinical opinion in 8.8% (this study) to 10% [17] of women supports tissue biopsy in the absence of imaging techniques sufficiently mature to replace pathology assessment. Given the consistency demonstrated between metastases at all sites [8], the site selected for biopsy and ER, PR, and HER2 analysis may now be considered less critical than once believed [31,32]. The resource implications for surgical or interventional imaging-guided biopsy and pathology assessment of biopsy material need to be balanced with the more rational use of therapy based on tumor receptor status.

Although prior therapies were not associated with a change in receptors [14] (loss of ER or PR was confirmed as the most common change in receptor status [14-17]), changes from ER-negative to ER-positive in 2.2% (by Allred score) and PR-negative to PR-positive in 12 patients (8.8%) were observed. Thus, failure to biopsy recurrent disease may deny such patients potentially effective treatment with endocrine therapy.

The clinical impact of these changes in receptor was reflected by the physicians who accrued patients to this study and who considered that the information from ER, PR, and HER2 assessment of the locoregional recurrence or metastatic disease would change the therapies offered in 17% of patients, emphasizing the potential of biopsy of breast cancer recurrence to positively influence therapeutic decision making [3,4,16,17]. Whether similar considerations apply to other epithelial cancers such as colorectal and ovarian cancer [33,34] is uncertain given the consistency in expression of some proteins between primary and metastasis in these cancer types.

## Conclusions

This large prospective study has demonstrated that the management of relapsed breast cancer should include tissue sampling to avoid misdiagnosis of 1 in 12 patients, to confirm the diagnosis of recurrent breast cancer, to identify switches of ER, PR, or HER2 status in the locally recurrent or metastatic breast cancer, and to influence the planned treatment for 1 in 6 patients.

## Abbreviations

BRITS: Breast Recurrence In Tissues Study; CI: confidence interval; ER: estrogen receptor; FFPE: formalin-fixed paraffin-embedded; FISH: fluorescence *in situ *hybridization; HER2: human epidermal growth factor type 2; IHC: immunohistochemistry; PR: progesterone receptor; TMA: tissue microarray.

## Competing interests

The writing committee (AMT, LBJ, PQ, AS, JAD, and CAP) has received educational grant funding and clinical trial funding from AstraZeneca (London, UK). EA is an employee of AstraZeneca. The authors declare that they have no other competing interests.

## Authors' contributions

AMT, EA, and JAD contributed to the design of the study and helped to draft and revise the manuscript. LBJ and CAP helped to carry out the histological studies and to draft and revise the manuscript. PQ performed supplementary statistical analyses and helped to draft and revise the manuscript. AS helped to draft and revise the manuscript. All authors read and approved the final manuscript.
